# Preemptive Levetiracetam Decreases Postoperative Pain: A Double-Blind, Randomised, Control Trial

**DOI:** 10.7759/cureus.33281

**Published:** 2023-01-02

**Authors:** Dipali Singh, Sourabh Kumar, Bharati LNU, Shio Priye, Jay Prakash

**Affiliations:** 1 Department of Anaesthesiology, Rajendra Institute of Medical Sciences, Ranchi, IND; 2 Department of Critical Care Medicine, Rajendra Institute of Medical Sciences, Ranchi, IND

**Keywords:** analgesic, preemptive, fentanyl, pain, levetiracetam

## Abstract

Background

Previously many studies have found the use of anti-epileptic drugs such as pregabalin, carbamazepine, and gabapentin in pain management. In addition, levetiracetam (LEV), an effective anti-epileptic drug, has shown analgesic effects in animal models. We aimed to evaluate the effect of oral LEV as pre-emptive analgesia in patients who underwent laparoscopic cholecystectomy under general anaesthesia and postoperative fentanyl requirements.

Material and methods

Forty-two patients of the American Society of Anaesthesiologists (ASA) grade I and II of either gender posted for elective laparoscopic cholecystectomy surgery were included in this double-blind, randomised, placebo-controlled study. Patients were divided into two equal groups of 21 each to receive either tablet LEV 500 mg or a matching placebo tablet, given 1 hour before surgery. Postoperative pain was assessed by a visual analogue scale (0-100 mm), where 0 meant no pain and 100, worst pain. In addition, patients received IV fentanyl as rescue analgesia during the first 24 hours of the postoperative period.

Results

Nineteen patients in the LEV group and 20 in the placebo group completed the study. Patients in the LEV group had significantly lower pain scores at all time intervals except 0 hours and reduced fentanyl consumption postoperatively in the first 24 hours (p<0.05). Side effects were comparable in both groups.

Conclusion

A single, preoperative dose of oral LEV 500 mg significantly decreases post-surgical pain and fentanyl demand as rescue analgesia in elective laparoscopic cholecystectomy.

## Introduction

Elimination and treatment of postoperative pain continue to be a major challenge in the postoperative area and plays a crucial role in the early mobilization and well-being of the post-surgical patient. Multimodal analgesia techniques that include pre-emptive analgesia can be used successfully in preventing postoperative pain. The reasoning behind pre-emptive analgesia is that antinociceptive treatment started before surgery is more beneficial in reducing postoperative pain rather than treatment started in the early postoperative period [1]. After elective laparoscopic cholecystectomy, pain is the most familiar complaint [2].

Levetiracetam (LEV) is a second-generation anti-epileptic drug (AED) that belongs to the pyrrolidone family, a class of drugs with a wide range of action. Experimental evidence indicates that LEV provides antihyperalgesic effects in inflammatory, chronic, and neuropathic pain models [3]. Although, the specific mechanism of action of LEV has yet to be fully elucidated. It is a structural analogue of piracetam with a favourable pharmacokinetic and tolerability profile. The analgesic activity of LEV has not been studied as extensively as that of gabapentin. However, it is well established that some anticonvulsants (e.g., carbamazepine and gabapentin) are beneficial in treating neuropathic pain. Moreover, recent meta-analyses have shown that perioperative administration of gabapentinoids (gabapentin and pregabalin) effectively reduce postoperative pain and opioid requirement [4]. Similarly, a single preoperative dose of gabapentin and pregabalin is useful for postoperative pain after laparoscopic cholecystectomy [5,6]. This study was planned to investigate the role of a single dose of LEV given preoperatively to reduce postoperative pain and fentanyl requirement in patients undergoing elective laparoscopic cholecystectomy.

## Materials and methods

After institutional ethical committee approval and CTRI registration (CTRI/2018/06/014404), written informed consent was taken from all patients. Forty-two adults (between 18 and 60 years of age), ASA I and II of either sex scheduled to undergo elective laparoscopic cholecystectomy, were included in this prospective, randomised, double-blinded, placebo-controlled study. Patients with alcohol abuse, known hypersensitivity to the study drug, significant respiratory, cardiac, renal or hepatic dysfunction or on chronic pain medication, anticonvulsant or antidepressant drugs, and uncooperative patients were excluded. In addition, patients who received analgesics 48 hours prior to the operation were not included in the study.

Patients were enrolled per inclusion criteria and randomly allocated into two equal groups of 21 patients using a computer-generated random number. The allocation was concealed in an opaque envelope and was only opened by a junior resident who was not participating in this study. Randomly assigned patients received oral LEV or matching placebo was given one hour prior to surgery. Patients were induced with propofol 2 µgkg-1, fentanyl 2 µgkg-1, and rocuronium 1 µgkg-1 for anaesthesia and endotracheal intubation. Patients were maintained with a mixture of oxygen and nitrous oxide in the ratio of 1:1 and isoflurane and a non-depolarising muscle relaxant. After surgery, patient reversal was achieved with glycopyrrolate 0.01 µgkg-1 and neostigmine 0.05 µgkg-1, and the patient was extubated. All patients who were enrolled in this study were counselled one day before about the visual analogue pain scale. Postoperative surgery pain was recorded at an interval of 0, 0-4, 4-8, 8-12, and 12-24 hours on the visual analogue scale (VAS; 0-100 mm) at rest. Pain assessment was done in the post anaesthesia care unit (PACU) (0 hours) and then every hour for up to 24 hours till the study ended. From collected pain score data, the highest pain scores during the time intervals of 0, 0-4, 4-8, 8-12, and 12-24 hours were evaluated for statistical analysis. Postoperative analgesia regimen included opioid analgesic with fentanyl injection 1 mcg/kg whenever the pain score was more than 40 mm on the VAS scale. As the onset of action of fentanyl is within 3-5 minutes, patients quickly get relieved. However, when the VAS score was more than 40 mm, the opioid was repeated after 1 hour. The total fentanyl requirement in the first 24 hours as rescue analgesia was recorded. The acuteness of postoperative pain and the requirement of fentanyl in the postoperative area were considered primary outcomes. Secondary effects were the incidence of side effects such as postoperative nausea and vomiting (PONV) and sedation. The intensity of PONV was graded on a four-point ordinal scale (0, no nausea or vomiting; 1, mild nausea; 2, moderate nausea; and 3, severe nausea with vomiting). All patients with PONV of a grade of more than 2 received ramosetron 0.3 mg IV. The Ramsay sedation scale (1- anxious and agitated, or restless; 2- cooperative, oriented, and tranquil; 3- responds to command only; 4- asleep but has a brisk response to a light glabellar tap or loud auditory stimulus; 5- asleep, has a sluggish response to a light glabellar tap or loud auditory stimulus; and 6- asleep, no response) was used to assess the sedation [7]. Patients having a sedation scale of more than 4 were believed as sedated.

Statistical analysis

This study was designed to assess the pre-emptive effect of LEV on fentanyl consumption in patients undergoing laparoscopic cholecystectomy. To evaluate, we did a pilot study on ten patients. Based on these data, SD in the control group was 69.15 and in the study group was 48.44, with a mean difference of 90 based on fentanyl consumption. For the result to be statistically significant with an alpha error of 5% and power of 90%, the required sample size in each group was nine patients. Assuming the dropouts increase the number of participants, the sample size in each group was taken as 21 patients. Patient characteristics and other continuous data were analysed with an independent sample t-test. Mann-Whitney U-test was used to analyse the postoperative fentanyl consumption and VAS pain scores. The incidence of side effects was evaluated using Fisher’s exact test. A p-value of less than 0.05 was considered statistically significant. The package SPSS 21 (SPSS Inc. Chicago, IL, USA) was used for statistical analysis.

## Results

A total of 42 patients were assessed and received study drugs from June 2018 to January 2019. One patient from the control group and two from the study group were considered dropouts after initial randomisation due to conversion to open cholecystectomy, and therefore, they were not analysed for further statistical analysis. There was no statistical difference among the groups with regard to age, weight, sex, duration of anaesthesia, and duration of surgery (P>0.05) (Tables 1-2). Total fentanyl consumption and postoperative pain were significantly less in the LEV group compared to the placebo group, except at 0 hours. (P<0.05) (Table 2). Statistically, there was no difference in the incidence of sedation, PONV and anti-emetics drug requirements when compared between the groups (P>0.05) (Tables 3-4).

**Table 1:** Patient demographics presented as mean (SD) or numbers. No significant difference between groups by independent sample t-test (P>0.05). M: Male; F: Female; LEV: Levetiracetam.

Variables	Placebo (21)	LEV (21)
Age (year)	41.04 (12.09)	39.90 (14.49)
Weight (kg)	55.71 (7.14)	58.23 (6.17)
Sex (M/F)	11/10	11/10

**Table 2:** All variables expressed as median (interquartile range) and analysed by the Mann-Whitney U test. LEV: Levetiracetam; VAS: Visual analogue scale.

Variables	Placebo (20)	LEV (19)	P-value
Duration of anaesthesia (min)	75.50 (17.5)	70 (10)	0.44
Duration of surgery (min)	57.50 (13.8)	55 (17)	0.81
0 h (VAS)	30 (10)	30 (20)	0.066
0-4 h (VAS)	60 (37.5)	30 (30)	0.008*
4-8 h (VAS)	55 (20)	40 (30)	0.016*
8-12 h (VAS)	50 (10)	30 (20)	0.001*
12-24 h (VAS)	40 (10)	20 (10)	0.001*
Total fentanyl (μg) consumption in 24 hours	180 (55)	60 (100)	0.001*
*P <0.05 is statistically significant			

**Table 3:** Sedation score (Ramsay sedation score). No significant differences between the groups by Fisher’s exact test (P-value: 0.909).

Sedation score	Placebo (20)	LEV (19)
1	3 (15%)	2 (10.5%)
2	5 (25%)	7 (36.9%)
3	8 (40%)	7 (36.9%)
4	4 (20%)	3 (15.7%)
5	0	0
6	0	0

**Table 4:** Incidence of side effects, data presented as numbers. No significant differences between the groups by Fisher’s exact test (P-value: 0.799). LEV: Levetiracetam; PONV: Post-Operative Nausea and Vomiting

PONV	Placebo (20)	LEV (19)
No	14 (70%)	13 (68.4 %)
Mild	1 (5%)	2 (10.5 %)
Moderate	1 (5%)	2 (10.5 %)
Severe	4 (20%)	2 (10.5 %)
Received antiemetics	4	2

## Discussion

In this study, we detected that a single preoperative dose of LEV 500 mg was efficient in decreasing postoperative fentanyl consumption and postoperative pain in patients of elective laparoscopic cholecystectomy. After oral intake of LEV, rapid absorption takes place, achieving its peak concentration after 1.3 hours. Its bioavailability is >95%. It has a volume of distribution of 0.5-0.7 Lkg-1. The plasma concentrations increase in proportion to the dose over the clinically acceptable dose range from 500 to 5000 mg, and there is no sign of accumulation during multiple administrations. Within 24-48 hours, the blood concentration of the drug achieved a steady state. LEV elimination half-life is 6-8 hours. Approximately 34% dose of LEV is metabolized, and the rest 66% is excreted unmetabolized via urine. However, it is not metabolized in hepatic but primarily in blood by hydrolysis [8].

Cortes-Altamirano JL et al. [9] reviewed studies on LEV, which suggest different mechanisms of action for LEV; however, the pharmacodynamics of this drug has not been fully elucidated. These studies provide evidence of the following three primary molecular targets: SV2A protein, inhibition of Ca2+ N-type channels, and the neuromodulator action on GABA, 5HT, α2-adrenergic, and μ-opioidergic pathways. The pharmacokinetic properties, effectiveness, high tolerability, low interaction with other drugs, and various mechanisms of action proposed for LEV have generated significant interest. They have opened up novel avenues for clinical research [4]. The antihyperalgesic effect of LEV and its mechanism of action were studied by investigating the involvement of GABAergic, opioidergic, 5-hydroxytryptaminergic (5-HTergic), and adrenergic systems in a rat model of inflammatory pain in a study conducted by Micov A et al. [3]. The authors reported that the effect of LEV in antihyperalgesic involves the indirect activation of central and/or peripheral GABA pathways by augmenting GABAergic neurotransmission, decreasing noradrenaline levels via its α2-adrenergic action, as well as modulating 5-HT levels utilizing various 5-HT receptors. LEV also influenced the μ-opioidergic receptors because these also inhibit N-type Ca2+ channels in a voltage-dependent manner [3]. This animal model of inflammatory pain indicates that LEV could be an essential agent for treating inflammatory pain in humans. LEV did not show any analgesic effects in the tail-flick and hot plate tests but has been shown to induce antihyperalgesic effects in models of neuropathic pain in rats. These outcomes indicate that LEV stimulates an antihyperalgesic effect in two types of human neuropathic pain, indicating a therapeutic potential in neuropathic pain patients [10]. LEV has also been recently reported to reduce anaesthetic-induced hyperalgesia in rats [11]. Data on the analgesic activity of LEV in humans is very limited. The pre-emptive administration of LEV significantly and dose-dependently inhibited postoperative hyperalgesia to a thermal stimulus, measured by paw withdrawal latency, after incisional hind paw surgery in rats. While morphine, administered pre-emptively, had almost no effect. Conversely, postoperative (therapeutic) administration of LEV did not reduce postoperative hyperalgesia, while morphine was effective when given postoperatively [4]. Das SK et al. found that the single dose of LEV preoperatively did not decrease the postoperative pain or rescue analgesic requirements. However, the author used an injection of paracetamol 1g 8th hourly and tramadol as rescue analgesia. The study indicates that VAS was lower at 0 hours and 3 hours, similar to our study [12].

A study by Rossi S et al. found that LEV is well-tolerated, working usefully against central pain, thereby enhancing the quality of life in multiple sclerosis patients. However, in this initial study, the power to recognize a significant effect in the placebo group was reduced because of its size and baseline pain level. The author concluded that larger samples of patients required with a double-blind design are necessary to approve these results [13]. LEV significantly increases the pain tolerance thresholds, and it has an analgesic effect in the electrical sural nerve stimulation human pain model [14]. Rowbotham MC et al. have reported that LEV in an open-label pilot study relieved pain and allodynia in patients having postherpetic neuralgia [15]. Against pain conditions in animal models of hyperalgesia in previous studies, LEV has been found to be effective. It is safe, effective, and well-tolerated in humans suffering from various peripheral or central neuropathic pain conditions.

The limitations of our study are that we did not use patient-controlled analgesia (PCA). However, when the study was proposed, the literature was inadequate about the effect of LEV on postoperative pain. Therefore, we principally wanted to express whether there was a reduction in postoperative fentanyl consumption. As the number of patients enrolled was less in this study, further studies are suggested to focus on the LEV effect in postoperative pain and its role as ‘pre-emptive’.

## Conclusions

In conclusion, we found that preoperative administration of LEV 500 mg was effective in lowering postoperative fentanyl consumption in 24 hours in patients who underwent elective laparoscopic cholecystectomy. In addition, the side effects were not significant in either group. We, therefore, recommend that a single dose of LEV 500 mg before elective laparoscopic cholecystectomy reduces pain and consumption of fentanyl in postoperative period.

## References

[1] Kissin I (1996). Preemptive analgesia. Why its effect is not always obvious. Anesthesiology.

[2] Dubois F, Icard P, Berthelot G, Levard H (1990). Coelioscopic cholecystectomy. Preliminary report of 36 cases. Ann Surg.

[3] Micov A, Tomić M, Popović B, Stepanović-Petrović R (2010). The antihyperalgesic effect of levetiracetam in an inflammatory model of pain in rats: mechanism of action. Br J Pharmacol.

[4] Sliva J, Dolezal T, Prochazkova M, Votava M, Krsiak M (2008). Preemptive levetiracetam decreases postoperative pain in rats. Neuro Endocrinol Lett.

[5] Pandey CK, Priye S, Singh S, Singh U, Singh RB, Singh PK (2004). Preemptive use of gabapentin significantly decreases postoperative pain and rescue analgesic requirements in laparoscopic cholecystectomy. Can J Anaesth.

[6] Agarwal A, Gautam S, Gupta D, Agarwal S, Singh PK, Singh U (2008). Evaluation of a single preoperative dose of pregabalin for attenuation of postoperative pain after laparoscopic cholecystectomy. Br J Anaesth.

[7] Ramsay MA, Savege TM, Simpson BR, Goodwin R (1974). Controlled sedation with alphaxalone-alphadolone. Br Med J.

[8] Patsalos PN (2004). Clinical pharmacokinetics of levetiracetam. Clin Pharmacokinet.

[9] Cortes-Altamirano JL, Olmos-Hernández A, Bonilla-Jaime H, Bandala C, González-Maciel A, Alfaro-Rodríguez A (2016). Levetiracetam as an antiepileptic, neuroprotective, and hyperalgesic drug. Neurol India.

[10] Ardid D, Lamberty Y, Alloui A, Coudore-Civiale MA, Klitgaard H, Eschalier A (2003). Antihyperalgesic effect of levetiracetam in neuropathic pain models in rats. Eur J Pharmacol.

[11] Archer DP, Lamberty Y, Wang B, Davis MJ, Samanani N, Roth SH (2007). Levetiracetam reduces anesthetic-induced hyperalgesia in rats. Anesth Analg.

[12] Das SK, Choupoo NS, Momin K, Das N, Das H, Sohkhia WE (2015). A single preoperative dose of levetiracetam has no effect on pain or analgesic requirements after laparoscopic cholecystectomy. Eur J Anaesthesiol.

[13] Rossi S, Mataluni G, Codecà C (2009). Effects of levetiracetam on chronic pain in multiple sclerosis: results of a pilot, randomized, placebo-controlled study. Eur J Neurol.

[14] Enggaard TP, Klitgaard NA, Sindrup SH (2006). Specific effect of levetiracetam in experimental human pain models. Eur J Pain.

[15] Rowbotham MC, Manville NS, Ren J (2003). Pilot tolerability and effectiveness study of levetiracetam for postherpetic neuralgia. Neurology.

